# Diagnostic Ambiguity Caused by an Atypical e18a2 *BCR*::*ABL1* Transcript in a Chronic Myeloid Leukemia Patient

**DOI:** 10.1155/2024/9439134

**Published:** 2024-11-25

**Authors:** Thomas Pretzsch, Steve Progscha, Thomas Burmeister

**Affiliations:** ^1^Labor Berlin Charité-Vivantes, Berlin, Germany; ^2^MVZ Leipzig Mitte, Leipzig, Germany; ^3^Charité–Universitätsmedizin Berlin, Corporate Member of Freie Universität Berlin and Humboldt-Universität zu Berlin, Campus Virchow, Medizinische Klinik für Hämatologie, Onkologie und Tumorimmunologie, Berlin, Germany

## Abstract

We describe the case of a chronic myeloid leukemia (CML) patient with a rare atypical e18a2 *BCR*::*ABL1* transcript. The generation of this transcript was explained by a detailed molecular analysis, including the identification of both chromosomal breakpoints (*BCR*::*ABL1* on der(22) and *ABL1*::*BCR* on der(9)) at the genomic level. The use of a cryptic splice site in intron 1 of *ABL1* led to the generation of an in-frame *BCR*::*ABL1* fusion transcript. The diagnostic difficulties caused by this atypical variant and its implications for diagnostic routine are discussed.

## 1. Introduction

The chimeric *BCR*::*ABL1* fusion gene, which usually results from the chromosomal translocation t(9;22)(q22;q34), is the molecular hallmark of chronic myeloid leukemia (CML) [1]. Approximately 98% of CML patients exhibit *BCR*::*ABL1* mRNA transcripts with a fusion of either *BCR* exon 13 or 14 with *ABL1* exon 2. In approximately 2% of the cases, “atypical” transcript variants, mostly e1a2, e13a3, e14a3, or e19a2, are observed [2, 3]. Atypical transcripts, i.e., those other than e1a2, e13a2, and e14a2, are also found at similar frequencies in adult *BCR*::*ABL1*-positive acute lymphoblastic leukemia patients [4]. It is important to identify atypical variants since these affected patients also benefit from tyrosine kinase inhibitor therapy. We describe the case of a CML patient with such an atypical transcript variant who was initially misdiagnosed, which led to diagnostic confusion years later when the patient relapsed. The transcript variant, a very rare e18a2 variant, was molecularly characterized. The molecular findings are discussed in the context of previously reported similar cases. The implications for diagnostic routines are discussed.

## 2. Case Presentation

### 2.1. Patient History

The patient, a then 49-year-old male from Germany, consulted his general physician because of unwanted weight loss, night sweats, and a decrease in performance. The patient had previously been healthy, and he did not smoke, drink alcohol, or use illicit drugs. The physical examination revealed a splenomegaly of approximately 20 cm, and a blood count revealed normochromic, normocytic anemia (hemoglobin 6.1 mmol/L), a pronounced leukocytosis of 284/nL and thrombocytosis of 806/nL. The differential blood count revealed 7.0% blasts, 3.0% promyelocytes, 22.0% myelocytes, 12.0% metamyelocytes, 11.0% band forms, 37.0% segmented neutrophils, 2.0% eosinophils, 5.0% basophils, 0.0% monocytes, and 1.0% lymphocytes. The lactate dehydrogenase (LDH) level was elevated to 3.7 times the upper limit of normal.

A bone marrow test revealed hypercellularity with predominant slightly dysplastic myelopoiesis and an overall blast content of 5.5%. Bone marrow fluorescence in situ hybridization revealed a *BCR*::*ABL1* fusion in 96% of the nuclei, and the karyogram was 46,XY,t(9;22)(q34;q11)[25]. The RT–PCR analysis in an external laboratory revealed a PCR product approximately compatible in size with an e19a2 *BCR*::*ABL1* transcript (“micro” *BCR*::*ABL1,* or “p230” BCR::ABL1). Sequence analysis of the transcripts was not performed. A diagnosis of CML in chronic phase was thus firmly established, and treatment with first hydroxurea followed by imatinib 400 mg daily was initiated.

### 2.2. Molecular Monitoring and Relapse

The following years were uneventful. The peripheral blood count was normalized under continuous imatinib treatment and regular PCR examinations were performed on peripheral blood. These investigations were performed in a different laboratory, and the patient was seen by different physicians. Unfortunately, the *BCR*::*ABL1* transcript information was communicated incorrectly, and the patient was further on reported as having an “e15a2” *BCR*::*ABL1* transcript. A quantitative RT–PCR test for “major” (e13a2/e14a2) *BCR*::*ABL1* showed that the patient was initially weakly positive, and the transcript decreased to undetectable levels after the third year of treatment. After nine and a half years, the patient stopped taking imatinib on his own initiative, stating that he had problems swallowing the tablets. One and a half years later, he exhibited persistent leukocytosis of 15–20/nL with increased neutrophil, basophil, and monocyte counts and slightly increased LDH levels. The subsequent bone marrow examination revealed only slight morphologic alterations in the marrow, but fluorescence in situ hybridization revealed *BCR*::*ABL1* fusions in 45.5% (91 of 200) of the nuclei. However, RT–PCR for the classical “major” (e13a2/e14a2) transcripts was negative. A sample was then sent to the authors' laboratory for further investigations.

### 2.3. Renewed Molecular Analysis

In the following section all nucleotide (ncl) coordinates refer to the GRCh38.p14 primary assembly.

**Figure 1:**
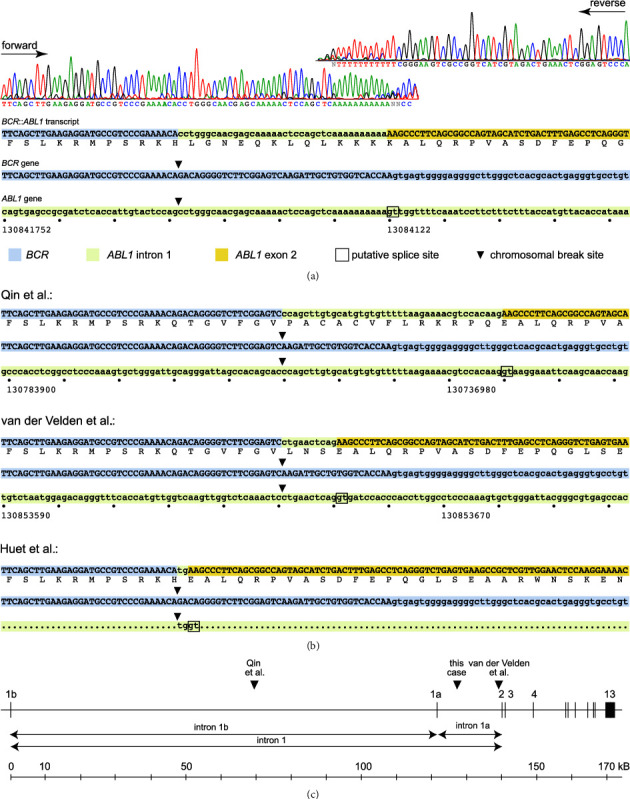
Sequence analysis of the detected e18a2 *BCR*::*ABL1* transcript and comparison with previously reported e18a2 *BCR*::*ABL1* cases. The exon sequences are UPPERCASE and the intron sequences are shown in lowercase. Chromosomal breakpoints are marked as triangles (▼) and putative splice sites are marked as open rectangles (□). (a) Sanger sequence chromatograms of the e18a2 *BCR*::*ABL1* transcript and genomic DNA sequences of the corresponding locations in *BCR* and *ABL1.* Near the *BCR*::*ABL1* fusion site, the sequence runs into a stretch of Ts at which the chromatograms end. (b) Sequences of the three previously reported e18a2 *BCR*::*ABL1* transcripts and their hypothetical chromosomal break positions and the splice sites used. (c) Location of the chromosomal breakpoints in the *ABL1* gene locus (hypothetical in the cases of Qin and van der Velden).

Multiplex RT–PCR revealed a PCR product of atypical size. The PCR product was sequenced, which revealed a truncated *BCR* exon 18 with the last 39 bp missing, and an intact *ABL1* exon 2. Between the exons, a 38 bp sequence with 100% homology to a part of *ABL1* intron 1a (chr9:130841784–130841821) was interposed. A subsequent PCR analysis of genomic DNA using the PCR primer pair ACTGTGCCCCTTCTCCCTACTGTA/TTTCCCTTTCCTGTCTCAACCTTTT revealed the der(22) chromosomal breaks on chromosome 22 after ncl 23310394 and on chromosome 9 after ncl 130841783 (Figure 1(a)). The generation of this in-frame *BCR*::*ABL1* fusion could be explained by the utilization of a cryptic splice site in *ABL1* intron 1a after ncl 130841821. The reciprocal der(9) chromosomal breakpoint was molecularly identified using the PCR primer pair TAGGCCACATTCAAAGCCGTCCT/CTACTGCTTCCAAGCCAGGTGG.

The patient insisted on only taking the smallest available tablets and thus ponatinib was started at a dose of 45 mg daily. After 3 months of treatment, no *BCR*::*ABL1* fusion was detected by FISH analysis. A specific quantitative RT–PCR assay with a forward PCR primer in *BCR* exon 18 (CAGGGAGTTCAGCTTGAAGAGGAT) was designed with PCR conditions otherwise as described previously [5], and the patient showed a relative *BCR*::*ABL1* transcript level of 0.23% in the peripheral blood after 12 months of ponatinib. The patient experienced a deep venous thrombosis in the left lower leg possibly associated with ponatinib use, after which edoxaban was started. The medication was, otherwise, well tolerated.

## 3. Discussion

Three similar but not identical e18a2 *BCR*::*ABL1*-positve CML cases were reported previously (Figures 1(b) and 1(c)). A simple direct fusion of *BCR* exon 18 with *ABL1* exon 2 would result in an out-of-frame and thus dysfunctional chimeric protein; thus, as in our case, more complex mechanisms were involved. In 2004, Qin et al. described the case of a 38-year-old male patient with an e18a2 *BCR*::*ABL1* transcript [6]. The transcript contained a truncated *BCR* exon 18 missing the last 19 nucleotides and an intact *ABL1* exon 2. A 40 bp sequence with sequence identity to a region in *ABL1* intron 1b (chr9: 130783950–130783989) was interposed between the exons. van der Velden et al. reported the case of a 16-year-old girl [5]. The e18a2 *BCR*::*ABL1* transcript showed the same truncation of *BCR* exon 18 as in the case reported by Qin, and 10 nucleotides with sequence identity to *ABL1* intron 1a (chr 9: 130853641–130853650) were interposed between the truncated *BCR* exon 18 and the intact *ABL1* exon 2. Huet et al. reported the case of a 44-year-old female patient with exactly the same break site in *BCR* exon 18 as in our case [7]. The transcript showed two inserted nucleotides (TG) between the truncated *BCR* exon 18 and the intact *ABL1* exon 2. All three e18a2 transcripts presented intact reading frames. No analysis of the genomic DNA was performed in any case (or it was unsuccessful), but the chromosomal breakpoints likely occurred after the same nucleotide in *BCR* exon 18 (chr22:23310413) in the first two cases and after nucleotides chr9:130783949 and chr9:130853640 in *ABL1* intron 1, respectively. The cryptic splice sites at chr9:130783990 and chr9:130853651 were likely used (Figure 1(b)). The patient in the second case was treated with imatinib and showed good clinical and molecular responses as revealed by a specially designed quantitative RT–PCR for e18a2 *BCR*::*ABL1*. In the third case, the chromosomal break in *ABL1* intron 1 putatively occurred two nucleotides before a cryptic splice site as illustrated in Figure 1(b). Since the sequence motif “TGGT” is common, the break site could not be precisely mapped.

In addition to these three cases, Sheng et al. described the co-occurrence of an out-of-frame e18a2 with an e19a2 *BCR*::*ABL1* transcript, likely caused by simple splicing of *BCR* exon 19 [8]. Fei et al. reported the case of a *BCR*::*ABL1*-positive acute myeloid leukemia patient with an e18a2 *BCR*::*ABL1* transcript, but the authors did not report any molecular details [9].

Atypical *BCR*::*ABL1* transcripts with chromosomal breaks in *BCR* exons are hypothesized to be more common than reported because many cases will remain unrecognized, i.e., misdiagnosed as typical transcripts, when the PCR product size resembles that of typical transcript variants. Since most CML patients respond well to tyrosine kinase inhibitors, this diagnostic error will often not become apparent and may be recognized only when the patient relapses. Assuming that chromosomal breaks in the *BCR* gene locus occur randomly, and that *in-frame* fusions with any of the first 19 *BCR* exons could result in functional *BCR*::*ABL1* transcripts and proteins, 2.5% of chromosomal breaks in the *BCR* gene locus are estimated to occur in exons (3325 bp of this region in the *BCR* gene are exonic and 129682 bp intronic). Most of these breaks in *BCR* exons will not result in *in-frame BCR*::*ABL1* transcripts and functional BCR::ABL1 proteins if the der(9) break is located in *ABL1* intron 1 unless the break occurs adjacent to a cryptic donor splice site that can be used to generate an *in-frame BCR*::*ABL1* transcript. The density and functionality of these cryptic splice sites in *ABL1* intron 1 are difficult to estimate. Notably, various in silico tools, such as *NetGene2 - 2.42* (https://services.healthtech.dtu.dk/services/NetGene2-2.42/) [10, 11], *NNSplice 0.9* (https://www.fruitfly.org/seq_tools/splice.html) [12], *MaxEntScan* (https://hollywood.mit.edu/burgelab/maxent/Xmaxentscan_scoreseq.html) [13], and *SpliceRover* (https://bioit2.irc.ugent.be/rover/splicerover) [14] did not predict a donor splice site in the unaltered *ABL1* intron 1 at chr 9:130841822, the apparent splice site identified in our case.

Aberrant splicing of the *BCR*::*ABL1* locus in CML is a well-known phenomenon [15, 16]. Shanmuganathan et al. reported relatively frequent aberrant splicing at low levels in classical CML patients with e13a2/e14a2 transcripts, with splice sites in *ABL1* intron 1 dispersed over a wide region (chr9:130716382 to chr9:130854066, hg38), covering nearly the entire *ABL1* intron 1 [17]. Various *BCR*::*ABL1*-positive cases in which the breakpoints occurred in a *BCR* exon and in *ABL1* intron 1 have been described. In these cases, cryptic splice sites in *ABL1* intron 1 were involved which led to the transcription of an in-frame chimeric transcript [3,18]. In other cases cryptic exons derived from the *ABL1* gene locus were interposed between out-of-frame *BCR* and *ABL1* exons, thus restoring the reading frame [19].

**Figure 2:**
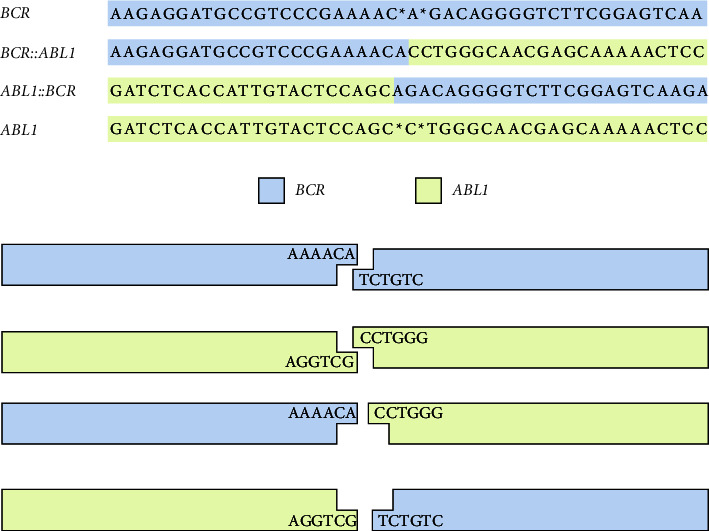
Breakpoint sequences and a hypothetical break mechanism. Nucleotide sequences of the *BCR*, *ABL1*, *BCR*::*ABL1*, and *ABL1*::*BCR* gene loci and a hypothetical model of the break and repair mechanism.

With respect to the chromosomal break mechanism, no sequence homologies were present at the break sites on chromosomes 9 and 22 (Figure 2). A duplication of one nucleotide was present on der(9) and der(22), which is compatible with a nonhomologous end-joining (NHEJ) repair mechanism [20].

## 4. Conclusion

Atypical *BCR*::*ABL1* transcripts are likely more frequent than reported because many will not be identified, if the PCR product sizes approximately match those of typical transcripts. The presented case illustrates that an incorrect characterization of the *BCR*::*ABL1* transcript variant at primary diagnosis may lead to problems that can manifest several years later. The well-established quantitative RT–PCRs for the common “major” and “minor” *BCR*::*ABL1* transcripts are able to detect certain rare, atypical transcripts, albeit with a low efficiency. For example, RT–qPCR for the e1a2 (“minor”) transcript may also detect the e6a2 transcript, and RT–qPCR for the e13a2/e14a2 (“major”) may detect the e19a2 variant. Because large PCR products are generated in these cases, the detection is inefficient and the observed transcript levels appear much lower (orders of magnitude) than they are. This carries a risk because patients are assumed to be in good remission, although this is not the case.

A lesson that could be learned from this case is that rare atypical *BCR*::*ABL1* variants detected by RT–PCR should be sequenced at first diagnosis to ensure that they are indeed the suspected variant. We also suggest that in cases such as ours, attempts should be made to identify the chromosomal breakpoint at the genomic level to obtain a better understanding of the aberrant splicing in *BCR*::*ABL1*. Second, plausibility checks should be performed for all quantitative measurements. An unusually fast decrease in transcript levels after the start of TKI therapy could be due to an atypical *BCR*::*ABL1* variant that is only inefficiently detected by the RT–qPCR assay used. A reassessment of the initial genetic analysis genetic should then be considered.

## Data Availability

All nucleotide sequences obtained in this work are accessible under the GenBank accession numbers PQ063830 (transcript), PQ063828 (genomic der(22) breakpoint) and PQ063829 (genomic der(9) breakpoint).
